# Multi-spectral fluorescent reporter influenza viruses (Color-flu) as powerful tools for *in vivo* studies

**DOI:** 10.1038/ncomms7600

**Published:** 2015-03-25

**Authors:** Satoshi Fukuyama, Hiroaki Katsura, Dongming Zhao, Makoto Ozawa, Tomomi Ando, Jason E. Shoemaker, Izumi Ishikawa, Shinya Yamada, Gabriele Neumann, Shinji Watanabe, Hiroaki Kitano, Yoshihiro Kawaoka

**Affiliations:** 1 Exploratory Research for Advanced Technology Infection-Induced Host Responses Project, Japan Science and Technology Agency (JST), Kawaguchi, Saitama 332-0012, Japan; 2 Division of Virology, Department of Microbiology and Immunology, Institute of Medical Science, University of Tokyo, Minato-ku, Tokyo 108-8639, Japan; 3 Laboratory of Animal Hygiene, Joint Faculty of Veterinary Medicine, Kagoshima University, Kagoshima 890-0065, Japan; 4 Transboundary Animal Distance Center, Joint Faculty of Veterinary Medicine, Kagoshima University, Kagoshima 890-0065, Japan; 5 Department of Pathobiological Sciences, School of Veterinary Medicine, University of Wisconsin-Madison, Madison, Wisconsin 53711, USA; 6 Laboratory of Veterinary Microbiology, Department of Veterinary Sciences, University of Miyazaki, Miyazaki 889-2192, Japan; 7 The Systems Biology Institute, Minato-ku, Tokyo 108-0071, Japan; 8 Sony Computer Science Laboratories, Shinagawa-ku, Tokyo 141-0022, Japan; 9 Okinawa Institute of Science and Technology Graduate University, Onna-son, Okinawa 904-0495, Japan; 10 Department of Special Pathogens, International Research Center for Infectious Diseases, Institute of Medical Science, University of Tokyo, Minato-ku, Tokyo 108-8639, Japan

## Abstract

Seasonal influenza A viruses cause annual epidemics of respiratory disease; highly pathogenic avian H5N1 and the recently emerged H7N9 viruses cause severe infections in humans, often with fatal outcomes. Although numerous studies have addressed the pathogenicity of influenza viruses, influenza pathogenesis remains incompletely understood. Here we generate influenza viruses expressing fluorescent proteins of different colours (‘Color-flu’ viruses) to facilitate the study of viral infection in *in vivo* models. On adaptation to mice, stable expression of the fluorescent proteins in infected animals allows their detection by different types of microscopy and by flow cytometry. We use this system to analyse the progression of viral spread in mouse lungs, for live imaging of virus-infected cells, and for differential gene expression studies in virus antigen-positive and virus antigen-negative live cells in the lungs of Color-flu-infected mice. Collectively, Color-flu viruses are powerful tools to analyse virus infections at the cellular level *in vivo* to better understand influenza pathogenesis.

## Abstract (web summary)

Animal models are important to study organismal immune responses to infection with influenza viruses. Here, Fukuyama *et al.* report a new generation of fluorescently labelled influenza viruses that facilitate the study of viral infections in animal models at cellular level.

## Graphical abstract

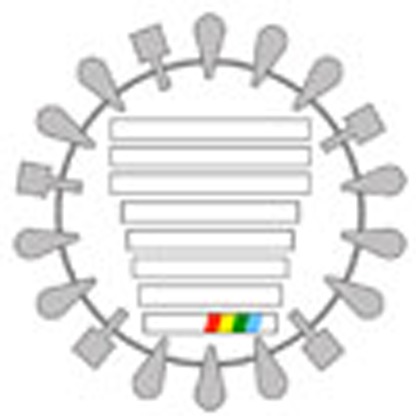

Influenza A virus is a respiratory pathogen that causes annual epidemics and sporadic pandemics^1^. Moreover, highly pathogenic avian H5N1 and the recently emerged H7N9 influenza viruses have caused an appreciable number of human infections with high mortality rates^2^,^3^. Influenza viruses infect respiratory epithelial cells and alveolar macrophages in mammalian hosts^4^. The host immune system recognizes the RNA genome of influenza viruses via cytosolic sensors^5^,^6^, which trigger innate immune responses that lead to the production of type I interferons (IFNs) and proinflammatory cytokines and chemokines^7^. Type I IFNs upregulate the production of antiviral proteins including myxovirus resistance (Mx), oligoadenylate synthetase (OAS) and interferon-stimulated gene 15 (ISG15)^8^. Dysregulation of the innate immune responses to influenza virus infection causes lung pathology mediated by infiltrating immune cells, including macrophages and neutrophils^9^,^10^. Although several studies have addressed host responses to influenza virus infections^11^, the mechanisms of influenza virus-induced pathology are still not fully understood.

To analyse the immune responses to influenza virus infection *in vivo*, viruses have been generated that expressed a fluorescent reporter protein^12^,^13^. However, these viruses were significantly attenuated^12^,^13^ and may not accurately reflect natural infections. Manicassamy *et al.*^14^ generated a GFP-expressing influenza virus, which they used to assess the route of antigen presentation on influenza virus infection^15^. However, the *GFP* gene was not stably maintained during replication in mouse lung or cultured cells, even though they isolated the GFP-expressing virus by repeated plaque purifications^14^.

Here we generated appreciably improved versions of fluorescent influenza viruses each of which stably expresses one of four different fluorescent proteins that can be monitored simultaneously. Plaque purification is not required for our strains to maintain the fluorescent expression. Using these viruses, we performed several studies to demonstrate the versatility of this novel tool set.

## Results

### Generation of ‘Color-flu’ viruses

**Figure 1:**
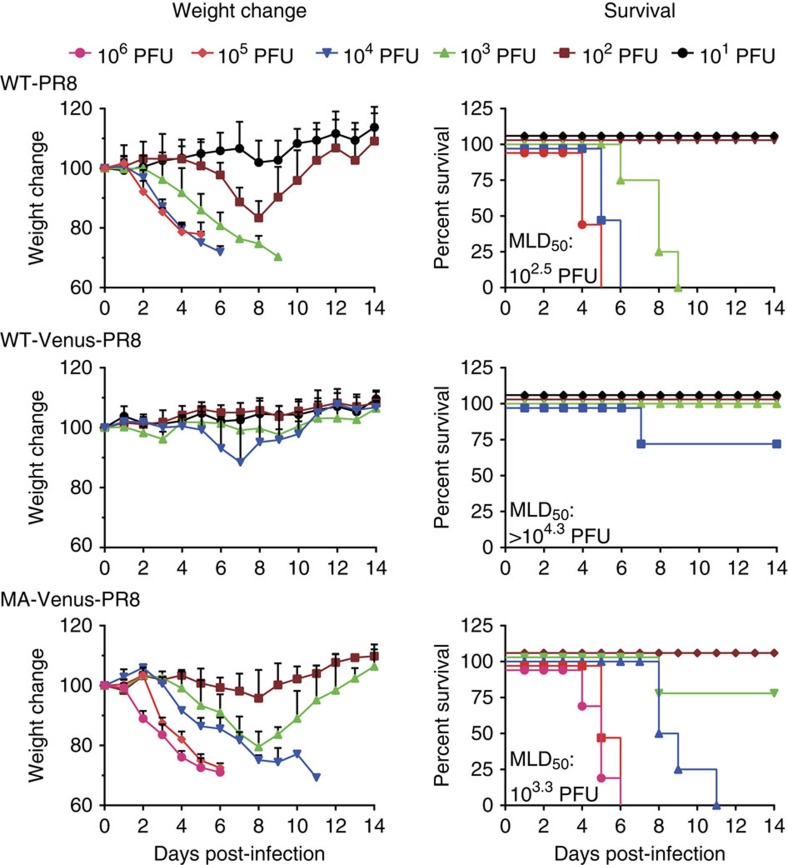
Characteristics of mouse-adapted Venus-PR8 in mice. Four B6 mice per group were intranasally inoculated with WT-PR8, WT-Venus-PR8 or MA-Venus-PR8. Body weight and survival of mice were monitored for 14 days.

To generate a fluorescent influenza virus expressing a reporter protein fused to the NS1 open reading frame, we chose Venus, a GFP variant with eight mutations including F46L, which improves chromophore formation and increases brightness compared with GFP^16^. As expected based on previous findings of attenuation for influenza viruses expressing reporter proteins^12^,^13^, the mouse pathogenicity of A/Puerto Rico/8/34 (PR8; H1N1) virus expressing Venus (WT-Venus-PR8) was substantially lower than that of wild-type PR8 (WT-PR8); the dose required to kill 50% of infected mice (MLD_50_) was more than 10^4.3^ plaque-forming units (PFU) for WT-Venus-PR8 compared with 10^2.5^ PFU for WT-PR8 (Fig. 1). We therefore serially passaged WT-Venus-PR8 in C57BL/6 (B6) mice. After six consecutive passages, we identified a variant (designated mouse-adapted (MA)-Venus-PR8; possessing a T-to-A mutation at position 380 of the hemagglutinin protein, and an E-to-D mutation at position 712 of the polymerase subunit PB2) with appreciably higher pathogenicity (MLD_50_=10^3.3^ PFU) compared with WT-Venus-PR8, although it was still slightly less pathogenic than the original PR8 virus (Fig. 1). To assess the replicative ability of MA-Venus-PR8 in mouse lungs, we intranasally infected B6 mice with 10^4^ PFU of MA-Venus-PR8 or WT-PR8. At all time points tested, the lung virus titres were similar for MA-Venus-PR8- and WT-PR8-infected mice (Table 1). To test the stability of Venus expression, we performed plaque assays using lung homogenate from infected mice and found that only one of 150 plaques on each of days 3, 5 and 7 post-infection (p.i.) was Venus-negative, attesting to the high genetic stability of Venus expression in this recombinant virus. In contrast, only 70% of NS1-GFP virus expressed the reporter protein^14^. The robust virulence and genetic stability of MA-Venus-PR8 indicate that this virus represents a highly attractive reporter system to visualize influenza virus-infected cells *in vivo*.

**Figure 2:**
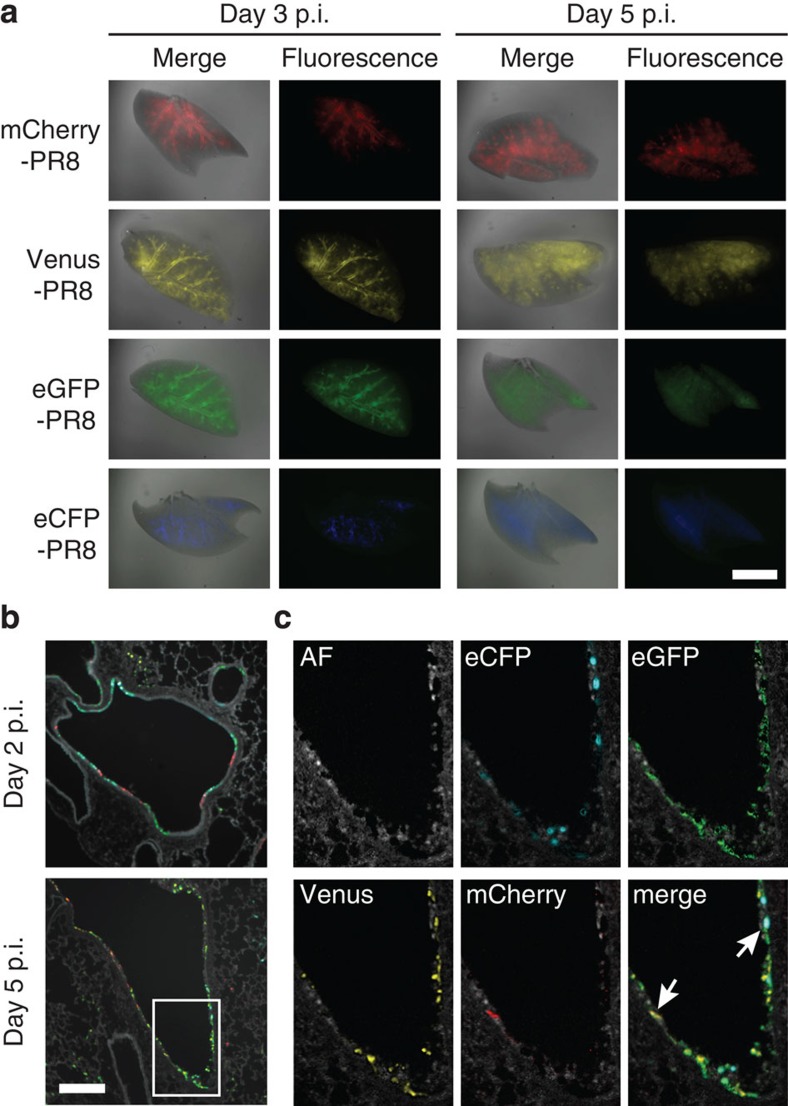
Distribution of Color-flu viruses in lungs. (**a**) Lung tissues were harvested from B6 mice at days 3 and 5 p.i. with Color-flu viruses (10^5^ PFU of MA-eCFP, eGFP, Venus and mCherry-PR8). Whole-mount images of transparent lung tissues were obtained by using a fluorescent stereomicroscope. Scale bar, 5 mm. (**b**,**c**) B6 mice were intranasally inoculated with a mixture of MA-eCFP, eGFP, Venus and mCherry-PR8 (2.5 × 10^4^ PFU per strain). (**b**) The sections of lungs at days 2 and 5 p.i. were analysed by using an inverted fluorescence microscope with a Nuance FX multispectral imaging system with inForm software. Scale bar, 100 μm. (**c**) Enlarged images of the indicated area in (**b**) were unmixed and separated into autofluorescence (AF), eCFP, eGFP, Venus and mCherry fluorescence. Arrows in the merged image indicate cells infected with different colour variants of Color-flu viruses.

To increase the versatility of fluorescent influenza viruses as imaging tools, we next generated additional MA-PR8 variants expressing different spectral GFP mutants, namely, eCFP (ex. 434 nm, em. 477 nm) and eGFP (ex. 489 nm, em. 508 nm)^17^. We also generated an mCherry variant (ex. 587 nm, em. 610 nm), which emits fluorescence at a longer wavelength than Venus (ex. 515 nm, em. 528 nm)^16^,^18^. These influenza viruses encoding the multi-spectral fluorescent reporter proteins were collectively named ‘Color-flu’. To determine the pathogenicity of Color-flu viruses, we compared the virus titres in mouse lung tissues and the MLD_50_ values of MA-eCFP, eGFP and mCherry-PR8 with those of MA-Venus-PR8 and MA-PR8. All of virus strains showed comparatively high replication in the lungs and the MLD_50_ values were similar among the Color-flu viruses (Supplementary Fig. 1 and Table 1). We also tested the stability of the fluorescent expression of the Color-flu viruses *in vivo* and *in vitro* by plaque assay. When we collected virus from the lungs of mice on day 7 p.i., the percentages of fluorescent-positive plaques were 98.0% (MA-eCFP-PR8), 100.0% (MA-eGFP-PR8) and 96.4% (MA-mCherry-PR8). We also measured the percentages of fluorescent-positive plaque in the sample from the culture medium of MDCK cells after 72 h p.i. and found them to be 100.0% (MA-eCFP-PR8), 99.2% (MA-eGFP-PR8) and 98.2% (MA-mCherry-PR8). In addition, we examined the stability of an NS1-fluorescent protein chimera in virus-infected cells by infecting MDCK cells with MA-Venus-PR8 virus and detecting NS1-Venus chimeric protein by using anti-GFP and anti-NS1 antibodies (Supplementary Fig. 2). We found that the NS1-Venus chimeric protein was not degraded until the time point we examined (that is, 12 h p.i.), indicating that the fluorescent signal is mainly emitted from the NS1-fluorescent protein chimera and not from degradation products in cells infected with Color-flu viruses. These findings indicate that the pathogenicity and stability of the Color-flu viruses were not affected by the different fluorescent reporter genes. To assess the expression of Color-flu viruses in mouse lungs, we collected lungs from B6 mice infected with each of the Color-flu viruses and processed them for visualization as described in the Methods section. All four colours were clearly visible in whole transparent lung tissue when analysed with a fluorescent stereomicroscope (Fig. 2a). Fluorescent signals were mainly seen in the bronchial epithelial layer at day 3 p.i. At day 5 p.i., fluorescent signals extended to the peripheral alveolar regions. These data indicate that all four Color-flu viruses are useful for analysing the distribution of influenza virus-infected cells in mouse lungs.

Next, we employed the Nuance spectral imaging system to test whether the fluorescent signals of all four Color-flu viruses could be detected simultaneously. Lung tissues were collected from B6 mice intranasally inoculated with a mixture of the four strains (2.5 × 10^4^ PFU each in a total volume of 50 μl). Analysis of lung sections obtained at days 2 and 5 p.i. showed that the fluorescent signals of all four Color-flu viruses were distinguishable from each other (Fig. 2b). At day 2 p.i., we found clusters of the same fluorescent colour in bronchial epithelial cells, suggesting local spread of the individual viruses. At this time point, a limited number of alveolar cells were infected. At day 5 p.i., we detected a cluster of alveolar cells expressing a single fluorescent protein, indicative of the initiation of infection with a single virus and its local spread (Fig. 2b). Interestingly, we also detected epithelial cells simultaneously expressing two or three fluorescent proteins, albeit at a low frequency, suggesting co-infection of these cells (Fig. 2c). To quantitatively analyse the co-infection in the bronchial epithelium, we utilized the inForm multispectral imaging software for automated user-trained tissue and cell segmentation, together with Nuance. We found that ~20% of epithelial cells were infected with multiple strains of Color-flu viruses in bronchus isolated from mice on day 2 p.i. (Supplementary Fig. 3). The ability to visualize cells co-infected with different influenza viruses *in vivo* is a major advance in technology and will allow us to provide novel insights into influenza co-infection and reassortment processes.

### The innate host response to Color-flu viruses

**Figure 3:**
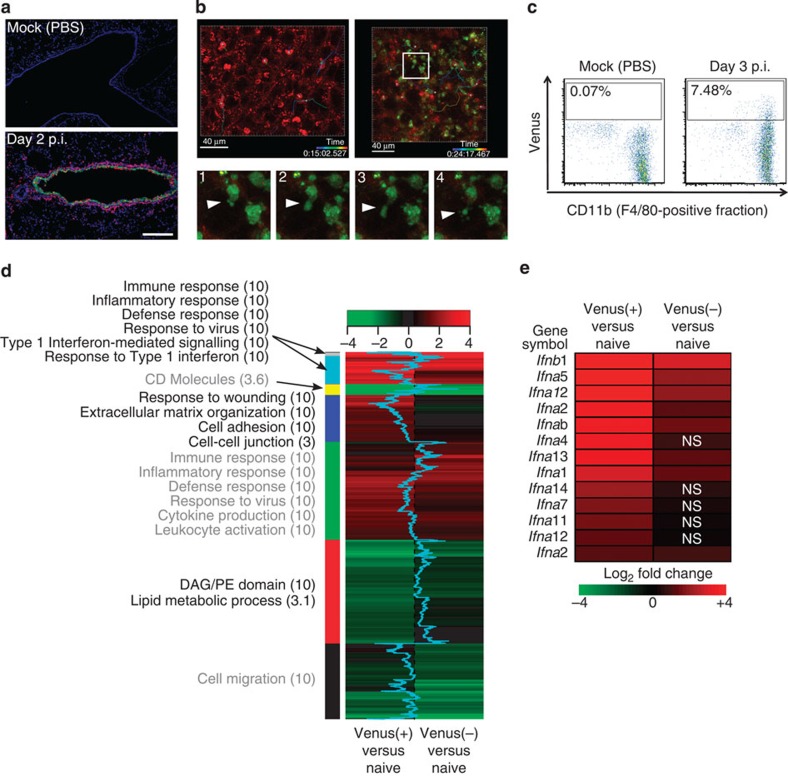
Analysis of lung macrophages. (**a**) Analysis of macrophage infiltration. Fixed lung sections of PBS-inoculated mice (mock) or mice infected with 10^5^ PFU of MA-Venus-PR8 at day 2 p.i. were incubated with an PE-anti-Mac3 antibody (red) and Hoechst dye (blue). The Venus fluorescent signal is shown in green. Scale bar, 200 μm. (**b**) Kinetics of the interactions between virus-infected cells and lung macrophages. Images of eGFP-positive cells (green) and CD11b+ macrophages (red) in lung tissue from naive B6 mice (upper left panel) and B6 mice on day 3 p.i. with 10^5^ PFU of MA-eGFP-PR8 (upper right panel) were obtained by using a two-photon microscope. The tracks of individual macrophages are indicated as coloured lines. Sequential images in the lower panel (1–4) show an enlarged view of the box in the upper right panel. Arrowheads indicate the blebbing of eGFP-positive cells. Scale bar, 40 μm. (**c**) Infection of macrophages by influenza viruses. Single-cell suspensions were obtained from lungs of PBS-inoculated (mock) mice or mice infected with 10^5^ PFU of MA-Venus-PR8 at day 3 p.i., The FACS analysis shows Venus expression from cells gated on F4/80 and CD45 expression levels. (**d**,**e**) Gene expression analysis. Total RNA was isolated from sorted macrophages of PBS-inoculated (naive) mice, and from sorted Venus-positive (Venus(+)) and Venus-negative (Venus(−)) macrophages of mice inoculated with 10^5^ PFU of MA-Venus-PR8 at day 3 p.i. (9 mice per treatment), and microarray analysis was performed. (**d**) Differentially expressed (DE) transcripts were identified by comparing gene expression levels in naive macrophages with those in Venus(+) and Venus(−) macrophages from infected mice. A heat map of the clustered transcripts for each condition is displayed, with enriched annotations and the enrichment scores for each cluster. A shift of the blue line in the heat map to the left or right indicates that the DE transcript is more highly expressed in Venus(+) or Venus(−) macrophages, respectively. (**e**) Heat map comparing expression levels of type I interferons (IFNs) between Venus(+) and Venus(−) macrophages. NS denotes not statistically significant between Venus(−) cells from infected animals and naive macrophages from uninfected animals.

We next tested the utility of Color-flu viruses for the analysis of host responses to infection. As macrophages are involved in innate immunity and acute inflammation in influenza virus-infected lungs, we examined lung sections stained with an antibody to macrophages (PE-Mac3) by using confocal microscopy. Macrophages infiltrated regions containing Venus-positive bronchial epithelial cells at day 2 p.i. of mice with MA-Venus-PR8 (Fig. 3a); by contrast, only a few Mac3-positive cells were detected in the alveoli of lungs from mock-infected animals. On the basis of this finding, we next employed live imaging to further study the interaction between influenza virus-infected epithelial cells and macrophages in mouse lungs. In the lung tissue of naive B6 mice, CD11b+ alveolar macrophages were detected by use of a two-photon laser microscope. Most of these macrophages did not migrate (that is, showed little movement) during the observation period (49 min; Supplementary Movie 1). In mice infected with MA-eGFP-PR8 virus, many CD11b+ macrophages appeared to be ‘attached’ to eGFP-positive epithelial cells (Supplementary Movie 2); moreover, some of these eGFP-positive epithelial cells exhibited blebbing similar to apoptotic cells (Fig. 3b). Interestingly, a number of CD11b+ macrophages quickly moved around the eGFP-positive epithelial cells, suggesting possible macrophage responses to inflammatory signals such as IFNs or chemokines. We also analysed the kinetics of the lung macrophages by tracking individual cells (Fig. 3b) and found that there were no obvious differences between the kinetics of macrophages in the naive lung and those in the infected lung. Our system can thus be used to monitor the *in vivo* interactions between virus-infected and immune cells.

A number of studies have assessed the transcriptomics and proteomics profiles of influenza virus-infected mice^19^,^20^. As these studies used whole lung samples, the results are the sum of virus-infected and uninfected cells, leading to the dilution of host responses and not allowing one to distinguish the profiles of infected cells from those of uninfected, bystander cells. As a first step to overcome this shortcoming, we sorted macrophages (known to be infected by influenza viruses (Fig. 3c)) from the lungs of mice infected with MA-Venus-PR8 on the basis of their fluorescent protein expression and performed microarray analysis. Macrophages isolated from the lungs of mice inoculated with PBS (naive macrophages) served as controls. In fluorescent-positive macrophages, 6,199 transcripts were differently expressed relative to naive macrophages. By contrast, in fluorescent-negative macrophages obtained from infected mice, only 4,252 transcripts were differentially expressed relative to the naive macrophages. This difference likely reflects differences in gene transcription induced by active influenza virus infection. However, it should be noted that the fluorescent-negative cell populations obtained from infected animals may have included infected cells in which the fluorescent signal had not yet been detected as would be expected at an early stage of virus infection. In fact, confocal microscopy revealed that it took 9 h to detect fluorescent protein expression in the majority of MDCK cells (Supplementary Fig. 4). Hierarchical clustering of differentially expressed transcripts, followed by functional enrichment analysis of each cluster, indicated that both fluorescent-positive and fluorescent-negative macrophages obtained from infected animals exhibit activation of pathways associated with the immune response, cytokine production and inflammation (Fig. 3d, green cluster). The upregulation of these pathways in the fluorescent-negative cells may have resulted from cell activation by IFN and cytokines released from infected cells, and/or from cells that were at an early stage of virus infection (as discussed earlier). Yet, a subset of enriched annotations, for example, type I IFN-mediated signalling (Fig. 3d, light blue cluster), included transcripts that were more highly expressed in fluorescent-positive macrophages. In addition, we observed that type I IFN genes were among the most upregulated transcripts in the fluorescent-positive macrophages (Fig. 3e). Taken together, this enhanced type I IFN activity is consistent with the suggestion that the fluorescent-positive cells had been infected, whereas the fluorescent-negative cells included both uninfected (but potentially ‘stimulated’) cells and cells at early stages of influenza virus infection. Indeed, it took at least 5 h to detect fluorescent protein expression after infection with Color-flu viruses, although all of the fluorescent proteins (that is, eCFP, eGFP, Venus, and mCherry) were detectable in the majority of cells by 9 h p.i. (Supplementary Fig. 4). These findings open new avenues in infectious disease research to compare gene expression (or other types of expression) patterns of reporter protein-positive cells with those of reporter protein-negative cells (but potentially stimulated by released cytokines and/or are at an early stage of infection).

### Avian influenza A (H5N1) virus expressing Venus protein

**Figure 4:**
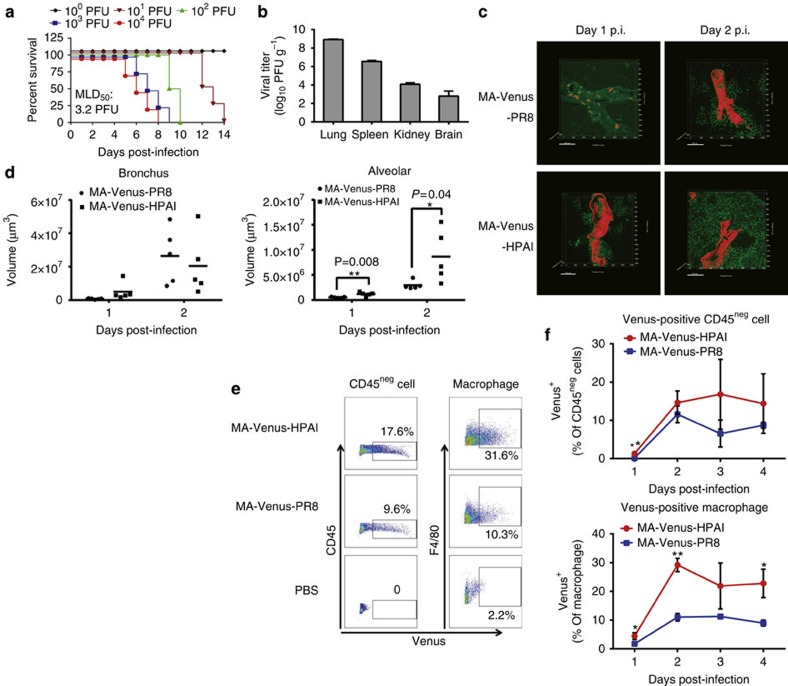
Characterization of MA-Venus-HPAI virus. (**a**) Four B6 mice per group were intranasally inoculated with MA-Venus-HPAI virus. Mouse body weight and survival were monitored for 14 days. (**b**) Lungs, spleens, kidneys and brains were harvested from B6 mice at day 3 p.i. with 10^5^ PFU of MA-Venus-HPAI virus. Virus titres of tissue homogenates were determined by use of plaque assays in MDCK cells. Each data point represents the mean±s.d. (*n*=3). (**c**,**d**) Lung tissues were harvested from B6 mice at day 1 and 2 p.i. with 10^5^ PFU of MA-Venus-HPAI and PR8. Images of transparent lung tissues with Venus-positive cells in the bronchus (red) and alveolar area (green) were obtained by using a two-photon microscope. Each data point represents the mean±s.d. (*n*=3). Statistical significance was calculated by using the Student’s *t*-test. (**d**) The distribution of Venus-positive cells was evaluated via volume analysis of the Venus-positive bronchus and alveolar area by using 3D images of the transparent lung tissues. (**e**,**f**) Cells were collected from lungs of B6 mice at days 1, 2, 3 and 4 p.i. with 10^5^ PFU of MA-Venus-PR8 or MA-Venus-HPAI virus, and stained for CD45, CD11b and F4/80. Venus expression in CD45-negative cells, and the Venus versus F4/80 staining profile gated on CD45-positive cells were analysed by flow cytometry. (**e**) A representative dot plot from day 2 p.i. is shown with the percentage of Venus-positive cells.

Finally, we tested whether the concept of mouse-adapted fluorescent influenza viruses could be applied to other influenza virus strains, such as highly pathogenic avian influenza A (H5N1) (HPAI) viruses, which are a research priority due to the threat they pose to humans. We generated an MA-Venus-HPAI virus based on A/Vietnam/1203/2004 (VN1203; H5N1), employing the same strategy used to create MA-Venus-PR8; we used the *PR8 NS* gene to express NS1-Venus chimeric protein because the *NS* gene did not contribute to the pathogenicity of VN1203 strain in mice^21^. The pathogenicity of MA-Venus-HPAI virus for B6 mice was comparable to that of VN1203, with MLD_50_ values for both viruses being less than 5 PFU (Fig. 4a and ref. 22). MA-Venus-HPAI virus also shared with other HPAI viruses the ability to spread systemically and replicate in various organs including the spleen, kidney and brain (Fig. 4b and ref. 22). Moreover, taking advantage of the strong fluorescent signal emitted by MA-Venus-HPAI virus- and MA-Venus-PR8-infected cells, we successfully constructed a three-dimensional image of an HPAI virus and PR8-infected bronchus as well as alveolar areas inside the lung tissues by using two-photon laser microscopy (Fig. 4c, Supplementary Movies 3–6)). This type of three-dimensional imaging analysis improves our understanding of the spatial distribution of influenza virus-infected bronchi. When we compared the distribution of virus-infected cells between HPAI virus- and PR8-infected lungs, we found that HPAI virus spreads from the bronchial epithelium to alveolar sites more quickly than did PR8 (Fig. 4c,d). We also found, by using flow cytometric analysis, that CD45-negative, non-hematopoietic cells and F4/80-positive macrophages more frequently expressed Venus in the lungs of mice infected with MA-Venus-HPAI virus than in the lungs of animals inoculated with MA-Venus-PR8 (Fig. 4e,f), supporting findings that H5N1 HPAI viruses induce more severe inflammatory responses in the lung than does PR8. Taken together, these findings demonstrate the utility of Color-flu viruses for comparative studies of influenza pathogenesis.

## Discussion

In this study, we established Color-flu viruses to study influenza virus infections at the cellular level. Color-flu viruses combine several improvements over existing systems, including robust viral replication, virulence, stable fluorescent protein expression and a set of four different colours that can be visualized simultaneously. We also demonstrated that Color-flu viruses are applicable to a different influenza virus strain. These improvements allowed global transcriptomics analyses of infected and bystander cells and, for the first time, live-imaging of influenza virus-infected cells in the mouse lung.

Previous versions of fluorescent influenza viruses^12^,^13^ including our original construct (that is, WT-Venus-PR8) were appreciably attenuated in mice. These attenuated fluorescent viruses may still be useful for identifying initial target cells. However, the immune responses elicited by these highly attenuated, non-lethal viruses most likely differ considerably from those of the mouse-lethal parent virus, making their use for pathogenesis studies problematic. Here, we solved this problem by passaging viruses in mice. This strategy proved to be successful for two different influenza virus strains, suggesting its broad applicability. A second drawback of previously tested fluorescent influenza viruses is the genetic instability of the added reporter protein^14^. We, however, found that >95% of virus plaques examined from mouse lung samples on day 7 p.i. expressed the reporter protein. We are currently studying the mechanism by which our mouse-adapted viruses stably express fluorescent proteins.

At present, Color-flu viruses cannot be monitored in live animals non-invasively because fluorescent reporter proteins must be within a ‘biological optical window (650–900 nm)’ to be detected for imaging of tissues in live animals using fluorescent probes^23^,^24^, and none of the fluorescent reporter proteins including mCherry, which has the longest emission among the reporter proteins of Color-flu, is inside this biological optical window. Several groups generated a luciferase reporter-expressing influenza virus that can be used to monitor virus replication in live animals^25^,^26^,^27^; however, this system needs systemic inoculation of substrate into the animals at every observation point. In addition, the resolution of their imaging system (based on the IVIS system) is not adequate for the analysis of cellular immune mechanisms *in vivo*, which we are able to achieve with our system.

Novel technologies for imaging analysis^28^ have enabled us to develop a set of four different influenza colour variants that can be distinguished from one another by using Nuance, hence allowing their simultaneous detection. In fact, our pilot study identified lung epithelial cells expressing two or three different fluorescent proteins (Fig. 2c, Supplementary Fig. 3). To our knowledge, this is the first visualization of mouse lung cells infected with more than one influenza virus strain. In future studies, these colour variants could be used to address long-standing questions in influenza virus research, such as the frequency of viral co-infections *in vivo*, which may be critical to better understand influenza virus reassortment and, hence, the generation of novel influenza viruses such as the pandemic viruses of 1957 (refs 29, 30), 1968 (refs 29, 30) and 2009 (refs 31, 32).

By employing our novel tool sets, we were able to detect influenza virus-infected cells in whole-lung tissues of mice, allowing us to observe the location and distribution of influenza viruses in the lung. Moreover, we were able to observe interactions of virus-infected epithelial cells with immune cells. Such studies will allow us to directly monitor influenza disease progression from acute bronchitis to severe viral pneumonia, which causes considerable morbidity and mortality in highly pathogenic influenza virus infections^33^,^34^.

In conclusion, Color-flu viruses in combination with advanced imaging technologies are a powerful and versatile tool to elucidate the mechanisms of influenza virus pathogenicity at the cellular level in animals.

## Methods

### Generation of Color-flu

The NS segments of PR8 fused with different fluorescent reporter genes including eCFP, eGFP, Venus and mCherry were constructed by overlapping fusion PCR as described previously^14^. In brief, the open reading frame (ORF) of the *NS1* gene without the stop codon was fused with the N terminus of fluorescent reporter genes via a sequence encoding the amino-acid linker GSGG. The fluorescent reporter ORFs were followed by a sequence encoding the GSG linker, a foot-and-mouth virus protease 2A autoproteolytic site with 57 nucleotides from porcine teschovirus-1 (ref. 14), and by the ORF of nuclear export protein (Supplementary Fig. 5). In addition, silent mutations were introduced into the endogenous splice acceptor site of the *NS1* gene to abrogate splicing^35^. The constructed NS segments (designated eCFP-NS, eGFP-NS, Venus-NS and mCherry-NS) were subsequently cloned into a pPolI vector for reverse genetics as described previously^36^. The plasmid encoding the Venus reporter protein was a kind gift from Dr A. Miyawaki (Laboratory for Cell Function Dynamics, RIKEN Brain Science Institute, Wako, Japan)^16^. WT-Venus-PR8 was generated by using the reverse genetics system as described previously^36^. As WT-Venus-PR8 pathogenicity and Venus expression levels were appreciably attenuated in mice, we serially passaged WT-Venus-PR8 in mice. After six passages, we obtained a variant (MA-Venus-PR8) with increased pathogenicity and strong Venus expression. A stock of MA-Venus-PR8 was generated in MDCK cells. As serial passage in animals typically results in virus populations composed of genetic variants, we recreated MA-Venus-PR8 by using reverse genetics. Likewise, MA-eCFP-PR8, -eGFP-PR8 and -mCherry-PR8 were generated with the same genetic backbone as MA-Venus-PR8. To generate a Venus-HPAI virus by reverse genetics, the NS segment of A/Vietnam/1203/2004 (H5N1; VN1203) was replaced with Venus-NS of PR8, and the virus was adapted to mice as described for MA-Venus-PR8. A stock of MA-Venus-HPAI virus was made in MDCK cells. The set of these influenza viruses carrying various fluorescent proteins was collectively termed ‘Color-flu’.

### Mouse experiments

Female, 6-week-old C57BL/6 (‘B6’) mice, which were purchased from Japan SLC, Inc. (Shizuoka, Japan), were intranasally inoculated with Color-flu viruses, at the dosages indicated in the figure panels, in 50 μl of PBS under sevoflurane anaesthesia, and body weights and survival were monitored for 14 days. Lungs were harvested from PBS-inoculated or Color-flu virus-infected mice for virus titration, flow cytometric analysis and histological experiments at the times indicated in the figure panels. All animal experiments were performed in accordance with the regulations of the University of Tokyo Committee for Animal Care and Use and were approved by the Animal Experiment Committee of the Institute of Medical Science of the University of Tokyo.

### Histology and cytology

Lungs were fixed in 4% paraformaldehyde (PFA) phosphate buffer solution. Fixed tissues were embedded in OCT compound (Sakura Finetek, Tokyo, Japan), frozen by liquid N_2_ and stored at −80 °C. Cryostat 6-μm sections were treated for 30 min with PBS containing 1% BSA (PBS-BSA) to block nonspecific binding, and then incubated with phycoerythrin (PE)-Mac3 (M3/84, BD Biosciences, San Jose, CA). To examine the cytology of the MDCK cells, we infected them with Color-flu virus and then fixed them in 4% PFA phosphate buffer solution. Nuclei were stained with Hoechst33342 (Invitrogen, Carlsbad, CA). Sections and cells were visualized by using a confocal microscope (Nikon A1Rsi, Nikon, Tokyo, Japan), controlled by NIS-Elements software. For quantitative multi-colour imaging analysis, the slides were visualized by use of an inverted fluorescence microscope (Nikon Eclipse TS100) with a Nuance FX multispectral imaging system with inForm software (PerkinElmer, Waltham, MA).

### Whole-mount imaging of lung tissue

Mice were killed and intracardially perfused with PBS to remove blood cells from the lung. The lungs were isolated after intratracheal perfusion with 4% PFA phosphate buffer solution. The lung tissues were cleared with SCALEVIEW-A2 solution (Olympus, Tokyo, Japan) according to the manufacturer’s instructions. Images were acquired by using a stereo fluorescence microscope (M205FA, Leica Microsystems, Wetzlar, Germany) equipped with a digital camera (DFC365FX, Leica Microsystems).

### Two-photon laser microscopy

A total of 10^5^ PFU of MA-eGFP-PR8 was intranasally inoculated into B6 mice. To label lung macrophages, 50 μl of PE-CD11b (M1/70, BioLegend, San Diego, CA) was injected intravenously to the mice at day 3 p.i. Thirty minutes after the antibody injection, the lungs of the mice were harvested. The kinetics of eGFP- and PE-positive cells in the lungs was imaged with a two-photon laser microscope (LSM 710 NLO, Carl Zeiss, Oberkochen, Germany). During the analysis, the lungs were maintained in complete medium (RPMI 1640 with 10% fetal calf serum) in a humid chamber (37 °C, 5% CO_2_). The data were processed with LSM software Zen 2009 (Carl Zeiss). For three-dimensional imaging of HPAI virus-infected lung tissues, B6 mice were intranasally inoculated with 10^5^ PFU of MA-Venus-HPAI virus and MA-Venus-PR8. The lung tissues were collected from the mice at day 1 and 2 p.i., and treated with SCALEVIEW-A2 solution (Olympus) to make tissues transparent as described above. Three-dimensional images of lung tissues were obtained from a two-photon laser microscope (LSM 710 NLO).

### Flow cytometric analysis and cell sorting

To obtain single-cell suspensions, lungs were dissociated with Collagenase D (Roche Diagnostics, Mannheim, Germany; final concentration: 2 μg ml^−1^) and DNase I (Worthington Biochemical, Lakewood, NJ; final concentration: 40 U ml^−1^) for 30 min at 37 °C by grinding the tissue through nylon filters (BD Biosciences). Red blood cells (RBCs) were lysed by treatment with RBC lysing buffer (Sigma Aldrich, St Louis, MO). To block nonspecific binding of antibodies, cells were incubated with purified anti-mouse CD16/32 (Fc Block, BD Biosciences, San Diego, CA). Cells were stained with appropriate combinations of fluorescent antibodies to analyse the population of each immune cell subset. The following antibodies were used: anti-CD45 (30-F11: eBioscience, San Diego, CA), anti-CD11b (M1/70: BioLegend), anti-F4/80 (BM8: eBioscience) and anti-CD11c (HL3: BD Biosciences). All samples were also incubated with 7-aminoactinomycin D (Via-Probe, BD Biosciences) for dead cell exclusion. Data from labelled cells were acquired on a FACSAria II (BD Biosciences) and analysed with FlowJo software version 9.3.1 (Tree Star, San Carlos, CA). To isolate Venus-positive and -negative macrophages from lungs, stained cells were sorted using a FACSAria II (BD Biosciences).

### Microarray analysis

Total RNA of sorted macrophages was extracted using TRIzol reagent (Life Technologies, Carlsbad, CA) and precipitated with isopropanol. RNA amplification was performed using the Arcturus Riboamp Plus RNA Amplification Kit (Life technologies) in accordance with the manufacturer’s instructions. RNA was labelled by using the Agilent Low Input Quick Amp Labelling kit, one colour (Agilent Technologies, Santa Clara, CA) and hybridized to the SurePrint G3 Mouse GE 8X60K microarray (Agilent Technologies). Arrays were scanned with a DNA Microarray Scanner with SureScan High-Resolution Technology, (G2565CA; Agilent Technologies), and data were acquired using Agilent Feature Extraction software ver. 10.7.3.1. (Agilent Technologies). Probe annotations were provided by Agilent Technologies (AMADID 028005). Probe intensities were background-corrected and normalized using the normal-exponential and quantile methods, respectively. The log_2_ of the intensities were then fit to a linear model that compared the groups of interest^37^. All reported *P* values were adjusted for multiple hypothesis comparisons using the Benjamini–Hochberg method. Transcripts were considered differentially expressed if there was at least a twofold change in the mean probe intensity between contrasts with an adjusted *P*<0.01. Hierarchical clustering was performed in *R*. The resultant gene clusters were then analysed with ToppCluster^38^ to identify gene annotations that were enriched in each cluster. The reported scores are the −log_10_ of the Benjamini–Hochberg adjusted *P* value.

### Western blot analysis

Whole-cell lysates of MDCK cells were electrophoresed through sodium dodecylsulfate polyacrylamide gels (Bio-Rad Laboratories, Hercules, CA) and transferred to a PVDF membrane (Millipore, Billerica, MA). The membrane was then blocked with Blocking One (Nacalai Tesque, Kyoto, Japan) and incubated with polyclonal rabbit anti-GFP (MBL, Nagoya, Japan), mouse anti-NS1 (188/5, a gift from Prof. H. Kida, Hokkaido Univ., Sapporo, Japan), rabbit anti-WSN (R309, prepared in our laboratory) or mouse anti-β-actin (A2228; Sigma-Aldrich), followed by HRP-conjugated anti-mouse or anti-rabbit IgG antibody (GE Healthcare, Waukesha, WI), respectively. After the membrane was washed with PBS-Tween, specific proteins were detected by using the ECL Plus Western Blotting Detection System (GE Healthcare). The specific protein bands were visualized by use of the VersaDoc Imaging System (Bio-Rad Laboratories).

## Author contributions

S.F., H. Katsura, D.Z., M.O., H. Kitano, G.N., S.W. and Y.K. designed the experiments and wrote the paper. S.F., H. Katsura, D.Z., M.O., T.A., I.I. and S.Y. performed experiments. S.F., H. Katsura, D.Z., M.O., T.A., I.I., J.E.S., S.W. and Y.K. analysed the data.

## Additional information

**Accession codes:** Microarray data have been deposited in the GEO data repository under the accession code GSE64473.

**How to cite this article**: Fukuyama, S. *et al.* Multi-spectral fluorescent reporter influenza viruses as powerful tools for *in vivo* studies. *Nat. Commun.* 6:6600 doi: 10.1038/ncomms7600 (2015).

## Supplementary Material

Supplementary Figures Supplementary Figures 1-5

Supplementary Movie 1 Time-lapse imaging of alveolar macrophages in naive mice. The lung tissues were harvested from naive B6 mice. Images of CD11b+ alveolar macrophages (red) were obtained by using a two-photon microscope at an excitation wavelength of 960 nm. This movie corresponds to the upper left panel in Figure 3b.

Supplementary Movie 2 Time-lapse imaging of macrophages and Venus-positive cells in mouse lungs infected with MA-eGFP-PR8. The lung tissues were harvested from B6 mice on day 3 p.i. with 105 PFU of MA-eGFP-PR8. Images of eGFP-positive cells (green) and CD11b+ macrophages (red) were obtained by using a two-photon microscope at an excitation wavelength of 850 nm. This movie corresponds to the upper right panel in Figure 3b.

Supplementary Movie 3 Three-dimensional images of MA-Venus-PR8-infected lung tissues. B6 mice were intranasally inoculated with 105 PFU of MA-Venus-PR8, and lung tissues were harvested on day 1 p.i. and cleared with SCALEVIEW-A2 solution. Venus signal in the bronchus (red) and alveolar area (green) from transparent lungs was detected by using two-photon microscopy at an excitation wavelength of 930 nm, and three-dimensional images were reconstructed by using Imaris software. This movie corresponds to the upper left panel in Figure 4c.

Supplementary Movie 4 Three-dimensional images of MA-Venus-PR8-infected lung tissues. B6 mice were intranasally inoculated with 105 PFU of MA-Venus-PR8; lung tissues were harvested on day 2 p.i. and cleared with SCALEVIEW-A2 solution. Venus signal in the bronchus (red) and alveolar area (green) from transparent lungs was detected by using two-photon microscopy at an excitation wavelength of 930 nm, and three-dimensional images were reconstructed by using Imaris software. This movie corresponds to the upper right panel in Figure 4c.

Supplementary Movie 5 Three-dimensional images of MA-Venus-HPAI virus-infected lung tissues. B6 mice were intranasally inoculated with 105 PFU of MA-Venus-HPAI virus; lung tissues were harvested on day 1 p.i. and cleared with SCALEVIEW-A2 solution. Venus signal in the bronchus (red) and alveolar area (green) from transparent lungs was detected by using two-photon microscopy at an excitation wavelength of 930 nm, and three-dimensional images were reconstructed by using Imaris software. This movie corresponds to the lower left panel in Figure 4c.

Supplementary Movie 6 Three-dimensional images of MA-Venus-HPAI virus-infected lung tissues. B6 mice were intranasally inoculated with 105 PFU of MA-Venus-HPAI virus; lung tissues were harvested on day 2 p.i. and cleared with SCALEVIEW-A2 solution. Venus signal in the bronchus (red) and alveolar area (green) from transparent lungs was detected by using two-photon microscopy at an excitation wavelength of 920 nm, and three-dimensional images were reconstructed by using Imaris software. This movie corresponds to the lower right panel in Figure 4c.

## Figures and Tables

**Table 1:** Replication and virulence of Color-flu in mice*.

**Virus**	**Mean virus titer (log**_**10**_ **PFU/g±s.d.) in the mouse lung on the indicated day p.i.**			**MLD**_**50**_ **(PFU)**
	**Day 3 p.i.**	**Day 5 p.i.**	**Day 7 p.i.**	
MA-eCFP-PR8	8.1±0.2†	8.0±0.1	6.3±0.1	10^3.0^
MA-eGFP-PR8	8.6±0.1	8.3±0.1	6.3±0.1	10^3.5^
MA-Venus-PR8	8.6±0.2	8.4±0.1	6.5±0.3	10^3.3^
MA-mCherry-PR8	7.7±0.3†	7.5±0.7	6.1±0.4	10^2.7^
WT-Venus-PR8	5.6±0.3†	5.3±0.3†	5.2±0.2†	>10^4.3^
WT-PR8	8.8±0.1	8.2±0.5	6.9±0.2	10^2.5^
MA-PR8	8.9±0.1	9.0±0.0	7.9±0.2†	10^2.3^

^*^B6 mice were inoculated intranasally with 10^4^ PFU of each virus in a 50 μl volume. Three mice from each group were killed on days 3, 5 and 7 p.i., and virus titres in the lungs were determined in MDCK cells.

^†^Statistical significance was calculated by using the Student’s *t*-test; the *P* value was <0.01 compared with the titres in the lungs of mice infected with WT-PR8 virus.
