# Supplementary material for: Multi-spectral fluorescent reporter influenza viruses (Color-flu) as powerful tools for in vivo studies
Source: Nat Commun. 2015 Mar 25;6:6600. doi: 10.1038/ncomms7600 (PMC4389232; doi:10.1038/ncomms7600)
Supplement: Supplementary Figures — 1-5 [file ncomms7600-s1.pdf]

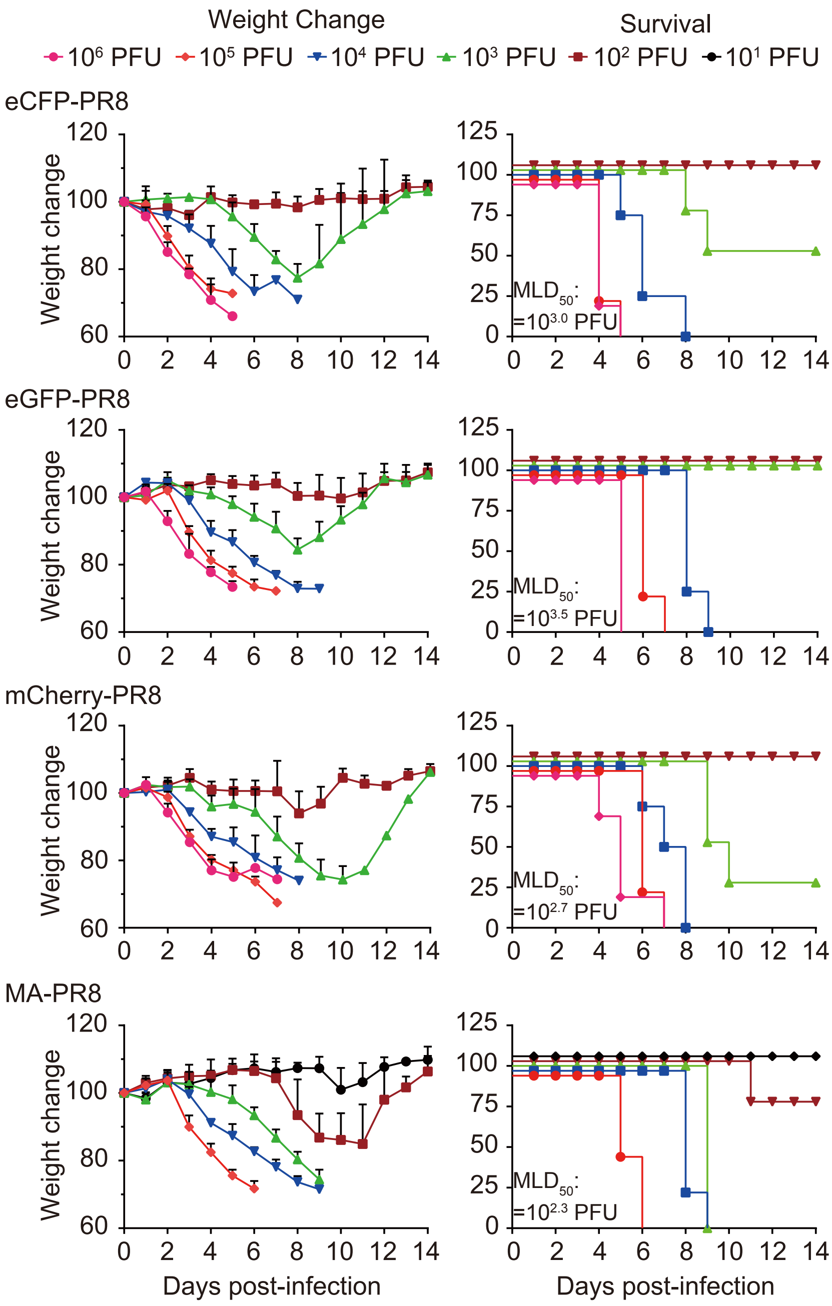

Supplementary Figure 1. Pathogenicity of Color-flu viruses in mice. Four B6 mice per group were intranasally inoculated with MA-eCFP, eGFP, mCherry-PR8, and MA-PR8. Body weight and survival were monitored for 14 days.

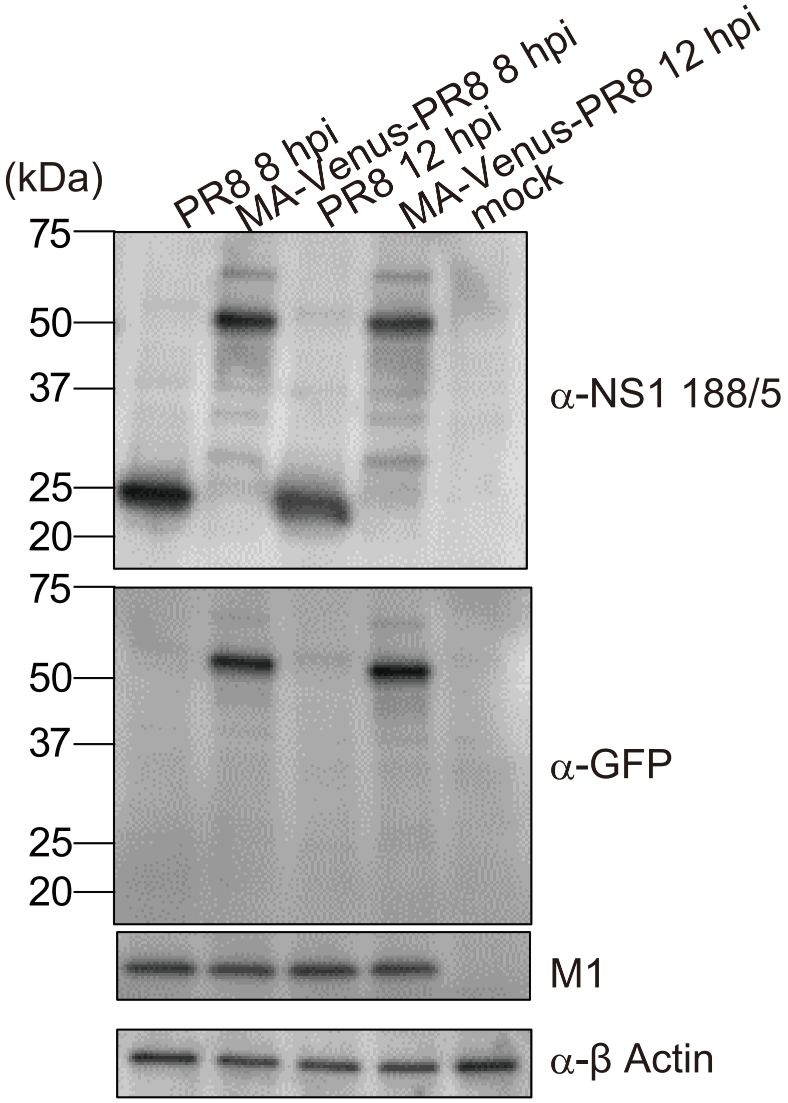

Supplementary Figure 2. Stability of an NS1-Venus chimera in MDCK cells. MDCK cells were infected with MA-Venus-PR8 or WT-PR8 at an MOI of 3. At 8 and 12 h after infection, NS1, Venus, M1, and β-actin were detected by means of western blotting.

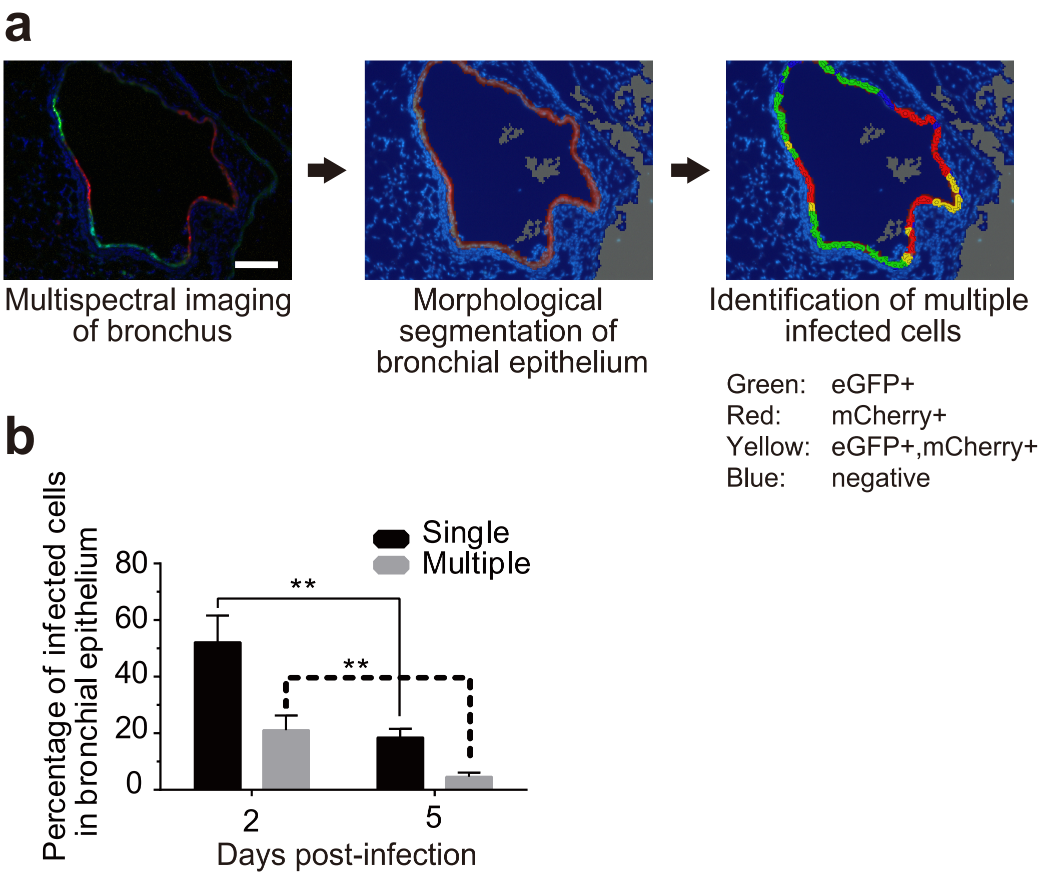

Supplementary Figure 3. Histological analysis of multiple infection in the lungs. B6 mice were intranasally inoculated with a mixture of MA-eCFP, eGFP, Venus, and mCherry-PR8 (2.5 x 10^4 PFU per strain). Lung sections were prepared from mice on days 2 and 5 p.i. (3 mice per time point). (a) Representative analysis of the area in the virus-infected bronchus from mice on day 2 p.i. by using an inverted fluorescence microscope with a Nuance FX multispectral imaging system and inForm software. (b) Cells infected with a single or multiple viruses in the bronchial epithelium. The statistical significance of differences was calculated using Student's t-test, and ***P* value was < 0.01 compared with a sample from day 5 p.i.

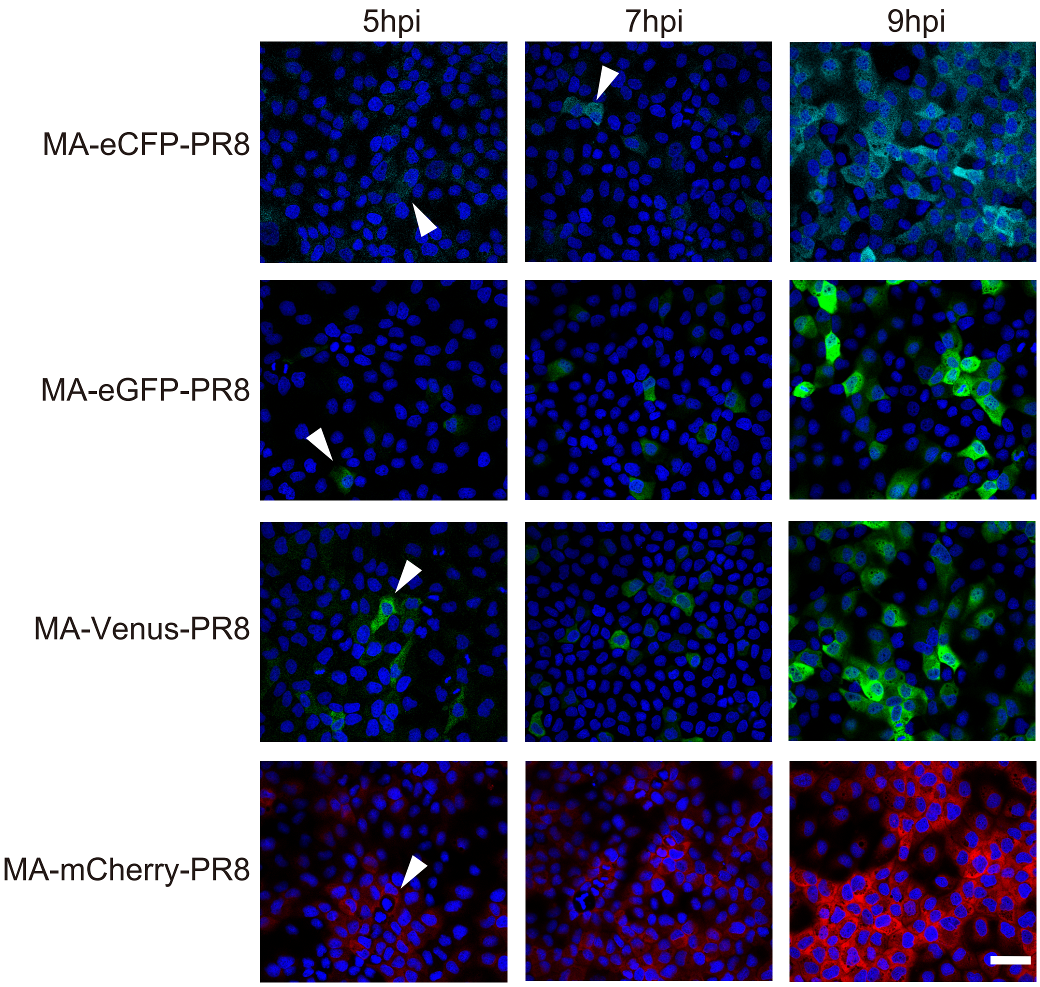

Supplementary Figure 4. Imaging of Color-flu virus-infected cells. MDCK cells were infected with Color-flu viruses at an MOI of 2. At 5, 7, and 9 h p.i., cells were visualized with a confocal microscopy. Arrowheads indicate cells expressing the fluorescent signal of each strain of Color-flu virus. Scale bar, 50μm.

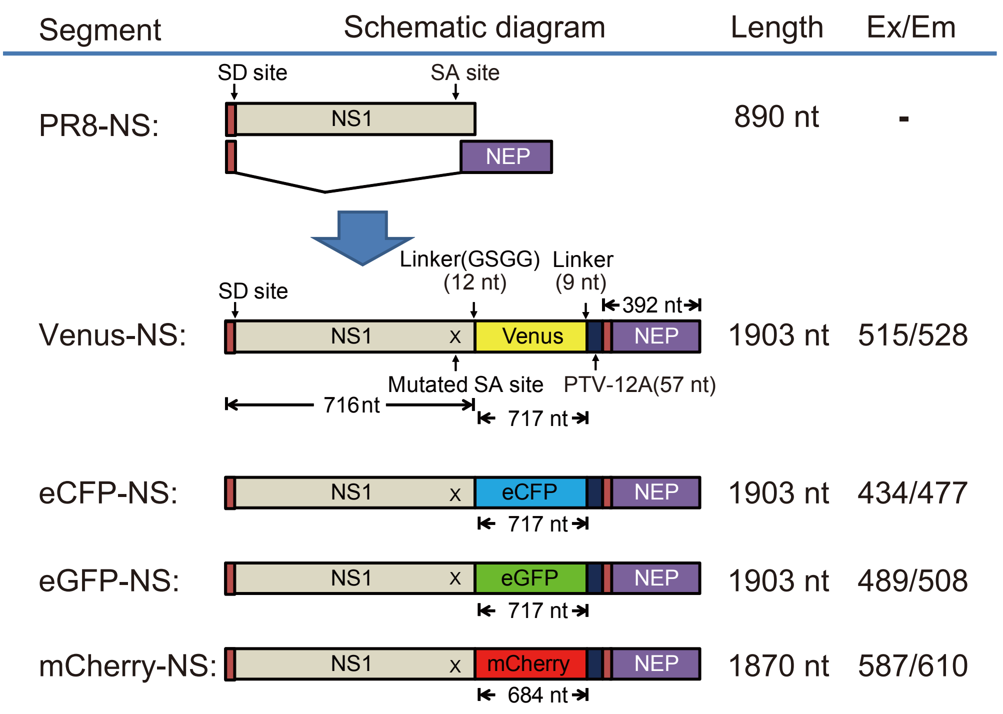

Supplementary Figure 5. Schematic diagrams of the NS segments of PR8 fused with different fluorescent reporter genes. The open reading frame (ORF) of the NS1 gene without a stop codon was fused with the N-terminus of fluorescent reporter genes (Venus, eCFP, eGFP, and mCherry) via a sequence encoding the protein linker GSGG. The fluorescent genes are followed by a sequence encoding the GSG linker, a foot-and-mouth virus protease 2A autoproteolytic site with 57 nucleotides from porcine teschovirus-1, and by the ORF of NEP. In addition, silent mutations were introduced into the endogenous splice acceptor site of the NS1 ORF to prevent splicing.
